# Patient-specific and interpretable deep brain stimulation optimisation using MRI and clinical review data

**DOI:** 10.3389/fnins.2025.1661987

**Published:** 2025-10-22

**Authors:** Apostolos Mikroulis, Andrej Lasica, Pavel Filip, Eduard Bakstein, Daniel Novak

**Affiliations:** ^1^Analysis and Interpretation of Biomedical Data, Department of Cybernetics, Faculty of Electrical Engineering, Czech Technical University, Prague, Czechia; ^2^Department of Neurology, First Faculty of Medicine, Charles University, Prague, Czechia; ^3^National Institute of Mental Health, Klecany, Czechia

**Keywords:** deep brain stimulation, optimisation, MRI, Parkinson’s disease, subthalamic nucleus, computational modelling

## Abstract

**Background:**

Optimisation of Deep Brain Stimulation (DBS) settings is a key aspect in achieving clinical efficacy in movement disorders, such as the Parkinson’s disease. Modern techniques attempt to solve the problem through data-intensive statistical and machine learning approaches, adding significant overhead to the existing clinical workflows. Here, we present a geometry-based optimisation approach for DBS electrode contact and current selection, grounded in routinely collected MRI data, well-established tools (Lead-DBS) and optionally, clinical review records.

**Methods:**

The pipeline, packaged in a cross-platform tool, uses lead reconstruction data and simulation of Volume of Tissue Activated (VTA) to estimate the contacts in optimal position relative to the target structure, and suggests optimal stimulation current. The tool then allows further interactive user optimisation of the current settings. Existing electrode contact evaluations can be optionally included in the calculation process for further fine-tuning and adverse effect avoidance.

**Results:**

Based on a sample of 174 implanted electrode reconstructions from 87 Parkinson’s disease patients, we demonstrate that our algorithm’s DBS parameter settings are more effective in covering the target structure (Wilcoxon *p* < 5e-13, Hedges’ g > 0.94) and minimising electric field leakage to neighbouring regions (*p* < 2e-10, g > 0.46) compared to expert parameter settings. Retrospective analysis of a limited subset (*n* = 50) predicts comparable improved motor outcomes with expert settings (g = 0.05–0.08, *p* = 0.09–1), suggesting potential for similar clinical efficacy, pending prospective validation.

**Conclusion:**

The proposed automated method for optimisation of the DBS electrode contact and current selection shows promising results and is readily applicable to existing clinical workflows. We demonstrate that the algorithmically selected contacts perform better than manual selections according to electric field calculations, without the iterative optimisation procedure.

## Introduction

Deep Brain Stimulation (DBS) is a treatment method for late-stage Parkinson’s disease (PD), aiming to minimise symptoms (Hariz and Blomstedt, 2022). It involves precise electrical stimulation that aims to modulate aberrant neural activity. Despite its clinical success, DBS parameters have to be optimised for each patient, leading to complex, empirical adjustments over several clinical visits. As a result, traditional DBS optimisation protocols are time-intensive, costly and burdensome for patients (Picillo et al., 2016; Volkmann et al., 2006).

Modern optimisation approaches attempt to resolve these issues through machine learning methods, such as autoencoder-based feature extraction and probabilistic models (Boutet et al., 2021; Qiu et al., 2024; Roediger et al., 2022). These methods excel in predicting the optimal stimulation parameters based on complex datasets, including functional magnetic resonance imaging (fMRI) response maps. However, their accuracy often comes at the cost of explainability, which may limit their adoption in clinical settings, where recommendations require a clear reasoning. Additionally, these techniques frequently rely on large-scale, high-dimensional datasets, which can be resource-intensive and clinically impractical. More specifically, the most recent approaches require machine learning models to be pretrained or additional pre-computed data; this requirement can be a discriminant analysis model (Boutet et al., 2021), a multilayer perceptron (Qiu et al., 2024), a collection of pre-compiled statistical models (Roediger et al., 2022), or fibre tract modelling and an additional, NEURON-based simulation step (Peña et al., 2018). For instance, imaging and electrophysiology-driven methods using connectomic modelling or advanced imaging modalities, such as functional MRI, have shown promise in guiding DBS programming but often require data not routinely collected in standard clinical workflows, including task-based or resting-state fMRI scans, connectomic reconstructions, or specialised electrophysiological recordings (Shah et al., 2023; Hines et al., 2024; Santyr et al., 2024).

Older works have explored biophysical modelling driven by imaging data to optimise DBS parameters, leading to explainable recommendations. The proposed pipelines for implementing this approach rely on either custom-coded FEM solutions (Anderson et al., 2018) or commercial visualisation software, used with fixed currents (Waldthaler et al., 2021), and operate in a manual or semi-manual manner. Similarly, sweet spot-guided algorithms have been developed to suggest contacts and amplitudes based on predefined empirical regions in the Subthalamic Nucleus (STN), using Volume of Tissue Activataed (VTA) approximations within Lead-DBS (Nordenström et al., 2022); however, these may depend on cohort-derived sweet spots and simplified spherical models, potentially limiting adaptability to individual patient anatomy.

In this study, we propose an open-source, cross-platform, GUI-based pipeline for stimulation parameter programming (i.e., electrode contact and current selection) that can be directly optimised through electric field calculations and its overlap with the targeted structure, based entirely on anatomical data from imaging and geometry principles. Our approach integrates magnetic resonance imaging (MRI) with a processing pipeline building upon well-documented DBS simulation tools to optimise DBS parameters in a manner that is minimally demanding in terms of data requirements, while incorporating input from personalised clinical evaluations.

Our processing pipeline utilises established processing tools: the Lead-DBS (Horn and Kühn, 2015) reconstruction of the implant, to derive the geometry of individual electrode contacts and the target structure of the stimulation, and OSS-DBS (Butenko et al., 2020) to perform fast, adjustable calculations of the reach of the electric field into the target structure. Clinical evaluations of the patient’s response to individual electrode contact and current selection during the initial clinical visit can be optionally integrated into the optimization procedure. Importantly, we prioritise simple user interaction, only requiring a reconstruction of the implant (in Lead-DBS format) and clinical evaluation of the electrode contacts and currents, if available.

We are evaluating our method using retrospective clinical data targeting the motor (dorso-lateral) subregion of the STN, which is a common target with demonstrated involvement in generating Parkinson’s Disease symptoms (Hacker et al., 2023; Vitek et al., 2022). This single-patient approach contrasts with prevailing machine learning-based methods by prioritising clinical utility, computational efficiency, with a lower setup and manual operation barrier compared to prior imaging-based methods, and robust integration with existing clinical data collection and basic reconstruction workflows.

## Materials and methods

### Patients and data

This study uses data from 104 patients with Parkinson’s disease who underwent bilateral implantation of deep brain stimulation electrodes (Medtronic 3,389, Medtronic B33 series, or Abbott 6,172), targeting the STN. In total, MRI data from 104 patients were processed, with 87 patients also having corresponding clinical review information and at least one hemisphere with documented stimulation settings. This yielded 174 implantation instances meeting all inclusion criteria for direct comparison.

All participants provided written informed consent to participate in the study upon enrolment. The study was approved by Ethics Committee of the General University Hospital in Prague (case number 59/18) and conducted in alignment with the Declaration of Helsinki. The data were sourced retrospectively from records of the iTEMPO department of the Neurology clinic, General University Hospital in Prague. For this dataset, the (average ± standard deviation) age at PD onset was 45 ± 8.6 (*n* = 64), and the age at DBS surgery was 56.2 ± 8.8 (*n* = 87). The surgeries for the DBS implants were performed between 2007 and 2023. A discrepancy between the numbers of patients with a PD onset record compared to the DBS surgery record is noted, due to incomplete reporting for some patients (hospital transfers).

We incorporated clinical evaluations of electrode contacts, done during initial stimulation setup at a clinical visit after implantation. The records indicated that the clinical review was performed in four contact group configurations per hemisphere: (i) individually activated edge contacts, (ii) separately tested middle non-directional contacts, or two groups of three radially arranged directional contacts in the case of directional leads. These groups were stimulated across incremental current levels ranging from 0.5 mA to 4.0 mA in 0.5 mA steps, with assessments focused on clinical improvement in rigidity, akinesia, and tremor, as well as thresholds for adverse effects.

Statistical analyses were conducted using the SciPy and Pingouin libraries, with Bonferroni-corrected Wilcoxon signed-rank tests for pairwise comparisons.

### MRI data processing

Pre-operative (3 T) and post-operative (1.5 T) MRI images (Nifti format) were imported into the Lead-DBS toolbox (Horn and Kühn, 2015), for atlas co-registration (using the SPM method; Friston et al., 2007), normalisation (using the ANTs method; Avants et al., 2008 with SyN nonlinear transform and mutual information metric), subcortical brainshift correction (Schönecker et al., 2009), and electrode reconstruction performed manually in Lead-DBS. The reconstructions were performed and validated by an expert neurologist.

All subsequent volume and coordinate processing was done after conversion back to the native patient space for each subject.

### Optimisation method

The proposed method, outlined below, consists of two steps: (i) contact selection based on the spatial configuration of the STN and the stimulation lead, and (ii) current selection, based on modelling of the Volume of Tissue Activated (VTA). In the second step, two variants of the method were evaluated: (a) geometry-based method only, and (b) a variant considering the clinical review with test stimulation in addition to the geometry-based method. The method is available as a standalone python-based GUI tool (Supplementary Figure S2; Mikroulis, 2025).

#### Contact selection

##### Geometry score

To identify the optimal contacts, we calculated their spatial relationship to the centre of mass of the motor subregion of the STN. Two geometric features were considered for each contact: (i) the Euclidean distance to the motor STN centroid, and (ii) the rotation angle between the contact and the centroid, relative to the electrode axis (for directional contacts). As these metrics differ in scale and units, they were independently ranked from lowest to highest across all contacts. The ranks were then summed to yield a geometry-based score for each contact $s_{\text{geometry},C}$ . For non-directional electrodes, and for edge contacts on directional electrodes where rotation is undefined, only distance was used; in such cases, a nominal angle of 90° was assigned to preserve consistency across contact types.

##### Clinical review-based score

The clinical review data only contained coarse contact groups (radially distributed directional contacts were evaluated as a single contact, making up a total of four coarse contacts in all types of electrodes). The clinical review thus evaluated four contact groups in all lead types.

The clinical evaluation data contained rigidity $s_{\text{rigidity},i,C}$ , akinesia $s_{\text{akinesia},i,C}$ , and tremor $s_{\text{tremor},i,C}$ scores, on a scale of integers ranging from zero (no symptoms) to six for each current setting at each contact group, along with notes where stimulation adverse effects occurred. In cases where a range between successive integers was noted as a score, it was transcribed as their average (for instance, a symptom score range of “2–3” was transcribed as “2.5” for the analysis). The total clinical score $s_{i,C}$ for every current step i and coarse contact C was calculated from the rigidity, akinesia, and tremor scores:


$$s_{i,C}=s_{\text{rigidity},i,C}+s_{\text{akinesia},i,C}+s_{\text{tremor},i,C}$$

An improvement is denoted by a decreasing clinical score sum, compared to the clinical score sum at the previous current step. The current steps starting from the first adverse effect occurrence were excluded from the evaluation.

The difference of the total clinical score was evaluated for successive current steps, i (up to the last adverse effect-free current step, I ) and scaled by the number of current steps divided with the upper bound of possible current steps ( $I_{\max}=8$ for our dataset) to prioritise faster improvements (with lower currents) and with the same linear scaling for all contacts, independently of the number of recorded current steps. The cumulative sum of the scaled differences was calculated for every current step, and scaled by the initial total clinical score (current step i=0 , with no current applied):


$$\delta_{I,C}=\frac{\sum_{i=1}^{I}(s_{i,C}-s_{i-1,C})(1-\frac{i-1}{I_{\max}})}{s_{0,C}}$$

with lower negative values of $\delta_{I,C}$ meaning greater improvement.

The best improvement for every contact was compared to the average of the improvements of all four contacts:


$$\beta_{C}=\frac{\min_{i}\delta_{i,C}}{\frac{1}{4}\sum_{C=0}^{3}\sum_{i}\delta_{i,C}}$$

The clinical review scaling factor κ was calculated by scaling the improvements of each contact to add up to 1, and calculating their ratio over ¼ (which would be their value if all four contacts achieved the same non-zero improvement):


$$\kappa_{\text{coarse},C}=\frac{\frac{\beta_{C}}{\sum_{C}\beta_{C}}}{\frac{1}{4}}$$

Since the clinical review performed in the clinical centre makes no distinction between the directional contacts ( c ) for every group of contacts C , the same value was used for all included directional contacts:


$$\kappa_{\text{directional},c}=\{\begin{array}{c} \begin{array}{c} \kappa_{\text{coarse},0},c=0\\ \;\kappa_{\text{coarse},1},c\in [1,3] \end{array}\\ \begin{array}{c} \;\kappa_{\text{coarse},2},c\in [4,6]\\ \kappa_{\text{coarse},3},c=7 \end{array} \end{array}$$

For the non-directional electrodes, the coarse evaluation contacts correspond to the physical contacts, so the same values were used:


$$\kappa_{\text{non}-\text{directional},c}=\kappa_{\text{coarse},C}$$

Depending on the electrode type (directional or non-directional), $\kappa_{c},$ was set to $\kappa_{\text{directional},c}$ or $\kappa_{\text{non}-\text{directional},c}$ respectively.

##### Combined score

For each contact, a coefficient based on the clinical scaling factor $\kappa_{c}$ , expressed as $1+(1-\kappa_{c})$ (to centre it around 1, with greater improvements approaching 0), was added to the geometry score $s_{\text{geometry},C}$ with 50% weight, according to:


$$S_{c}=\frac{1}{2}\cdot s_{\text{geometry},c}+\frac{1}{2}\cdot (1+(1-\kappa_{c}))\cdot s_{\text{geometry},c}$$

Based on the value of the combined score $S_{c}$ , the contacts with the two to three lowest scores were selected. The third contact was added for directional leads, only if (a) its score was tied with the second contact, or (b) if the score difference between the second and third was smaller than that between the first and second contacts and also smaller than the score of the top-ranked contact.

For the non-directional electrodes, the selection was restricted to the two best contacts. The second contact was added if its score difference from the first contact was smaller than the first contact’s score.

#### VTA modelling and overlap

The volume of activated tissue was calculated using OSS-DBS v2 (Butenko et al., 2020), at an electric field threshold of 200 mV/mm (Brown, 2017). The standalone OSS-DBS was preferred over the default Lead-DBS-integrated methods since it allows more flexibility in iterative settings modification for contacts and currents, and operates in the patient-specific native space, which eliminates discrepancies introduced by co-registration. For the simulated stimulations, the standard recommended settings were used (monopolar stimulation, 130 Hz, 60 μs pulse trains; Picillo et al., 2016; Volkmann et al., 2006). The VTA was calculated as a mesh by thresholding the electric field magnitude. The default solver iteration limit was increased to 5,000 steps, to avoid non-converging runs. The default OSS-DBS behaviour of generating a “success” file was disabled to allow multithreading.

The coordinates of the electrode contacts (including subcortical refinement/brainshift corrections) were retrieved from the pre-processed Lead-DBS reconstruction files. The DISTAL atlas (Ewert et al., 2018) was used, since it includes the histological labels necessary to calculate precise electric fields, as well as STN parcellation into the motor, associative and limbic subregions. Similarly, the coordinates of the STN subregions were retrieved from the pre-processed Lead-DBS native-space coordinates files. The electric fields were generated directly by OSS-DBS in VTK format.

To calculate VTA overlap with STN subregions, the coordinate sets provided by Lead-DBS were converted into convex hulls using the Delaunay method. Overlap metrics were computed as (i) the proportion of STN subregion mesh intersecting the VTA (motor, limbic and associative), and (ii) the fraction of VTA mesh intersecting the motor STN region.

#### Current selection

Next, the following current selection was performed for the set of contacts, pre-selected in the previous step, using VTA calculation and its overlap with STN subregions.

To estimate the stimulation current needed, an initial VTA was calculated for a 4 mA current ($I_{\text{initial}}$), which typically covers a substantial portion of the motor STN subregion and represents a typical upper-bound from a clinical perspective. The selected electric field threshold (200 mV/mm) was divided with the median electric field over the motor STN, to derive a scaling factor for the initial current accounting for conductivity variations in the medium: $I_{\text{suggested}}=I_{\text{initial}}(\frac{E_{\text{threshold}}}{E_{\text{median},\text{initial}.}})$

The median field magnitude was preferred over the average as a more robust measure for field intensity distributions with large spikes (for example, cases where the electrode may be located too close to the target region). The current was rounded to the closest 0.1 mA. For the single current selection, if the selected current was lower than 1 mA, 1 mA was selected, and clipped at 3.5 mA if it exceeded that value.

The final result consists of the contact and current selection, and the overlap of the VTA with motor and non-motor areas. An important output in judging the contact and current selection efficacy is the fraction of the VTA that is contained within the motor subregion. Additionally, we calculate the current-normalised values for these overlaps, to gauge the efficacy of the contact selection.

### A GUI tool for parameter setting

The aforementioned method is implemented as a Python-based GUI (Supplementary Figure S2) app (Mikroulis, 2025) and provides the recommended settings in CSV format. These results include the contact selections (for up to three directional contacts in Medtronic B33-series and Abbott 617x leads, by default), the current estimates for the 200 mV/mm median electric field threshold in the motor STN, and the corresponding overlaps with the STN subregions as fractions. Additionally, to facilitate exploration of the space of possible parameter settings by the user, the VTAs for four more currents (0.8, 1.6, 2.4, 3.2 mA) can be optionally calculated (for a total of up to six current—VTA pairs). The resulting parameters, including subregion overlaps, are interpolated using a cubic spline fit over the range of 0.5 to 4 mA for the three subregions of the STN and presented in an interactive window. An additional assisting tool highlights a range of current values to optimise motor STN coverage against VTA outside the motor STN subregion, limbic STN coverage, and associative STN coverage, using a selectable minimum percentage of the corresponding harmonic mean range (over the entire current range).

### Output evaluation

The contact and current settings, suggested by the proposed method, were compared against the clinical DBS settings used by patients. As most patients were followed longitudinally, often with multiple adjustments over time, only the earliest complete set of stimulation parameters was analysed to minimise confounding effects from disease progression, electrode drift, or current increases. For patients programmed using voltage-controlled stimulation, current values were derived using the recorded electrode impedance. In cases involving interleaved stimulation, the effective current was calculated as a weighted average based on the duty cycle duration, pulse duration, and frequency of each setting.

The contact selection between the two methods (algorithm-based and clinical settings) was compared with a Jaccard index. For each implanted electrode, the cross-method Jaccard index was calculated from the ratio of coinciding contacts selected by both methods over the set of all selected contacts by either method. For directional contacts, all the subcontacts were considered for this calculation.

### Outcome prediction

Motor symptom improvement was estimated using a subset of patients (*n* = 50) who underwent UPDRS-III evaluation of DBS efficacy within 3 years post-surgery. Of these, a smaller group (*n* = 18) had their first UPDRS-III assessment conducted during the same visit in which DBS settings were first recorded (6–12 months postoperatively), while the remaining patients had delayed assessments occurring 1 to 3 years after surgery. For each patient, the relative motor improvement was calculated using paired UPDRS-III scores with stimulation turned on and off during the same visit:


$$\frac{\text{scor}e_{\mathrm{DBS}:\mathrm{off}}-\text{scor}e_{\mathrm{DBS}:\mathrm{on}}}{\text{scor}e_{\mathrm{DBS}:\mathrm{off}}}$$

Two related variables were used to predict the improvement: (i) normalised motor-STN coverage as a measure of contact selection effectiveness, and (ii) current (mA) as a measure of stimulation strength affecting the size of the VTA.

A random forest regression model was trained using a maximum tree depth of seven splits, as performance plateaued beyond depth six and showed no further improvement at depths eight or nine. To reduce variance and stabilise predictions, the model was configured with 5,000 estimators.

## Results

### Output overview

**Figure 1:**
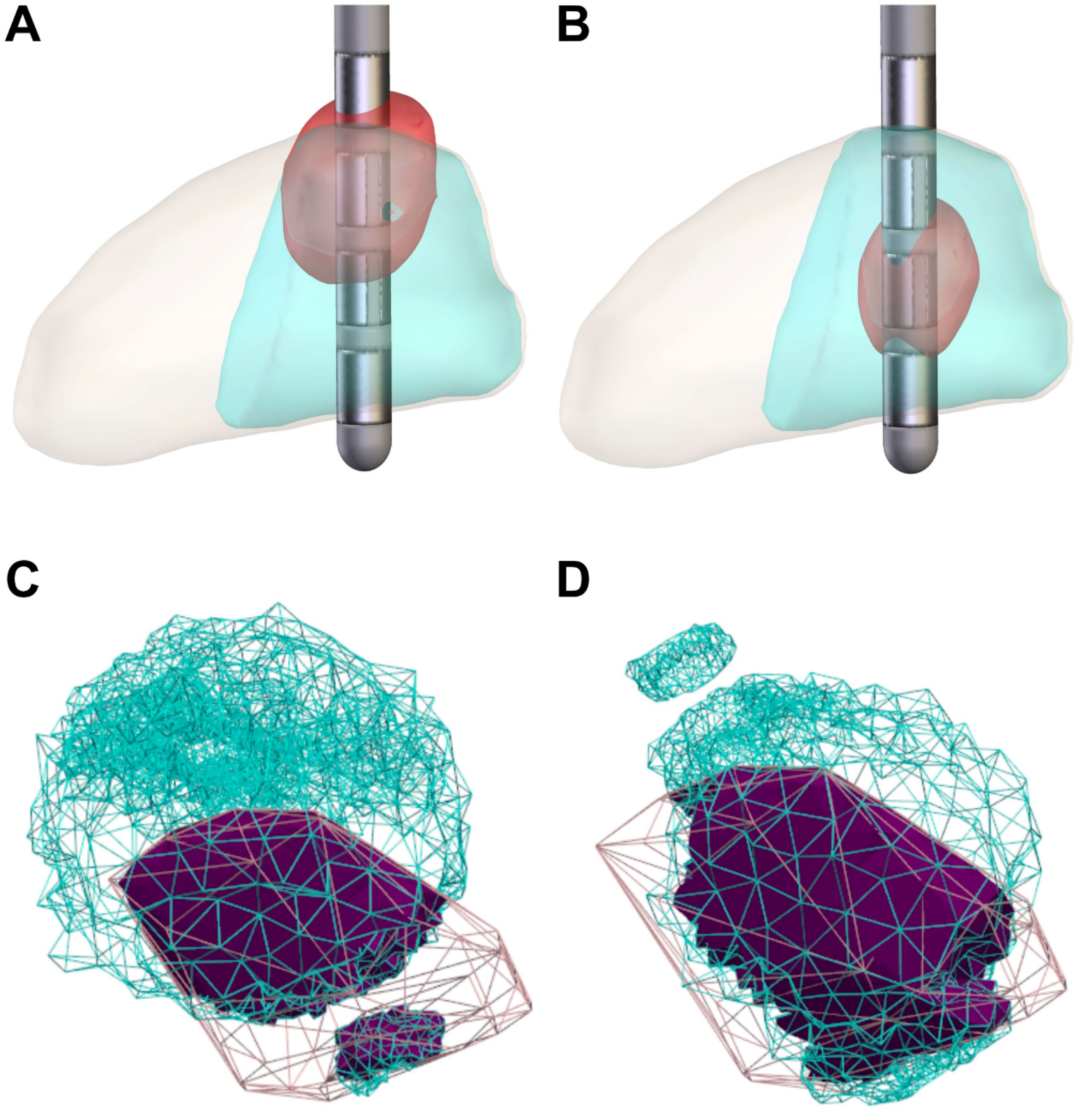
Visualisation of the VTA for clinically selected, patient settings and algorithm-selected settings. **(A,B)** Visualisation of the VTA approximation by Lead-DBS, using **(A)** patient settings and **(B)** algorithm-selected settings (red: VTA approximation, light orange: STN, cyan: motor subregion of the STN). **(C,D)** Visualisation of the VTA estimated by OSS-DBS at a minimum field intensity of 200 mV/mm, with the same settings as **(A,B)**, using **(C)** patient and **(D)** algorithm-selected settings (cyan: VTA estimate, pink: motor STN subregion, purple: overlap).

In total, the algorithm was evaluated on 174 implantations from 87 patients. Figure 1 shows examples of the resulting stimulation settings, visualized using VTA approximation in Lead-DBS (Figures 1A,B), or directly using the thresholded electric field data from OSS-DBS (Figures 1C,D, see 3D models in the Supplementary material).

### Comparison with clinical settings

**Figure 2:**
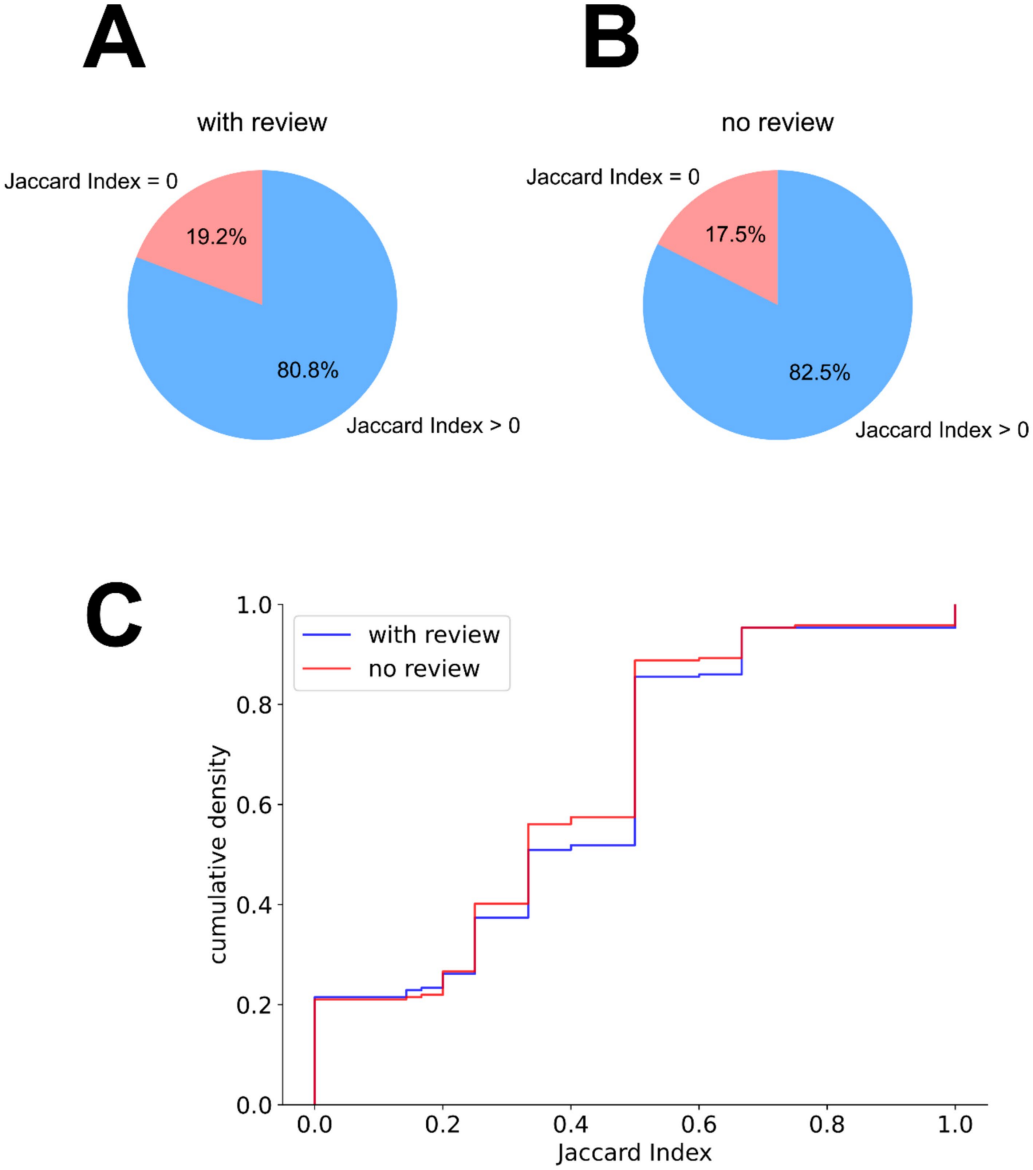
Contact selection similarity between expert-selected patient settings and algorithm-selected settings. **(A,B)** Partial overlap in the algorithm’s contact selection with the expert-selected contacts, based on the Jaccard index between these two contact sets per implant, **(A)** without the integration of clinical review information and **(B)** with the integration of the clinical review information. **(C)** Empirical cumulative distribution of the Jaccard indices between expert and algorithm contact selections, when using (blue) and not using (orange) the clinical review data.

The contact selection was assessed in comparison to the clinical settings, in two different scenarios: (a) without any clinical information, and (b) using clinical review data (weighted at 50%). The contact selection overlapped in approximately 80% of the cases (Figures 2A,B). To quantify the overlaps, for each implant, a Jaccard index was calculated between the expert-selected contacts and the algorithm-selected contacts (expressed as the intersection of the two contact sets over their union). This quantification showed slightly increased partial overlaps with the expert contact selections when the clinical review information was used (Figure 2C).

**Figure 3:**
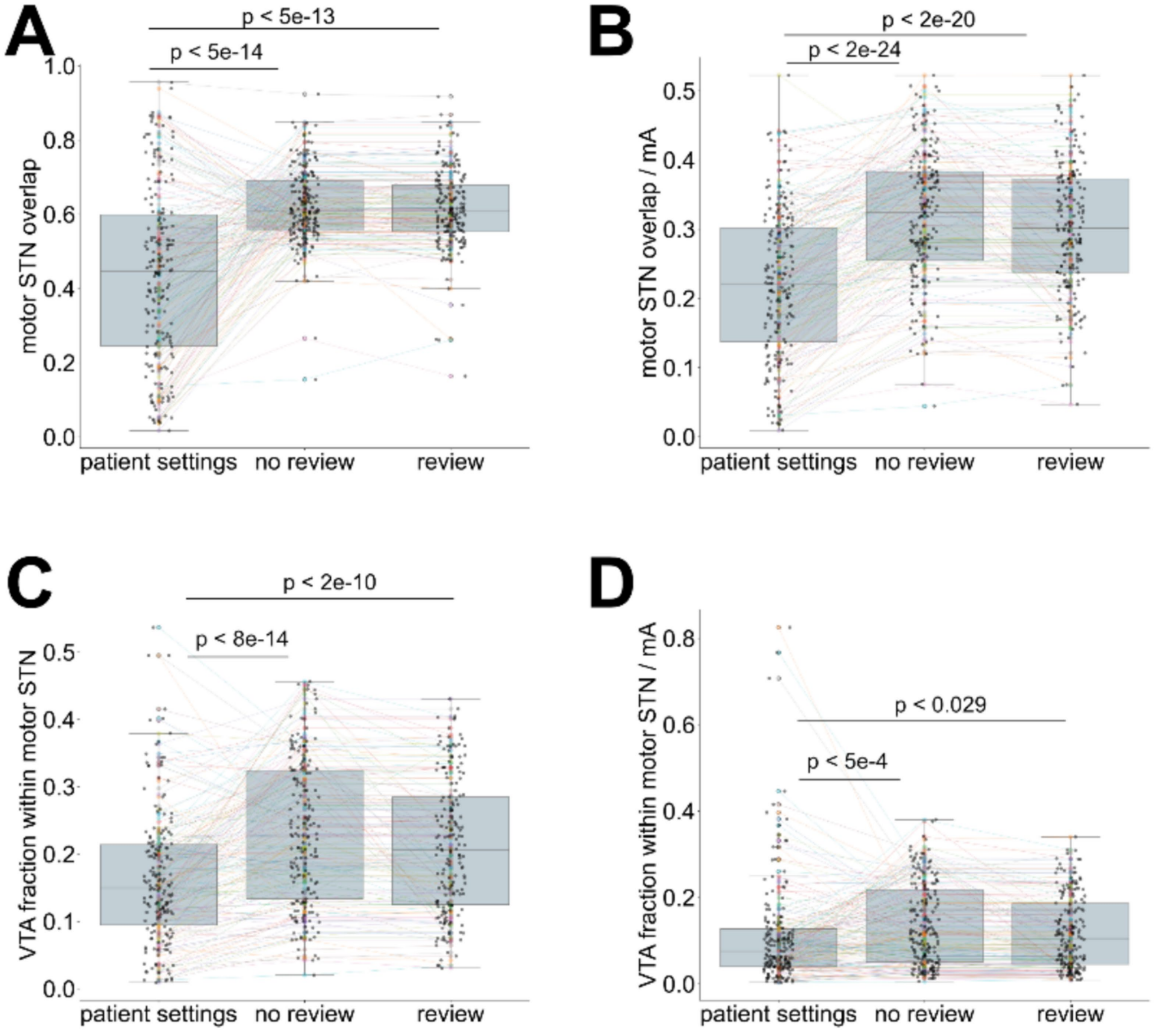
Comparison of calculated settings (with or without clinical lead contact review information) to the expert-selected patient settings. **(A)** coverage of the motor STN, using algorithm-selected settings (with and without clinical review integration) and expert-selected contacts (patient settings), **(B)** current-normalised coverage of the motor STN using algorithm-selected settings (contacts and currents—with and without clinical review integration) and expert-selected settings (patient settings), **(C)** fraction of the VTA contained within the motor STN using algorithm-selected settings (contacts and currents—with and without clinical review integration) and expert-selected settings (patient settings), **(D)** current-normalised VTA contained within the motor STN using algorithm-selected settings (contacts and currents—with and without clinical review integration) and expert-selected settings (patient settings). Wilcoxon test *p*-values are indicated for paired comparisons.

The VTAs generated with the algorithm-selected contacts and current outperformed the manual expert settings in terms of motor STN coverage (Hedges’ g > 0.94, Wilcoxon *p* < 5e-13) and VTA containment within the motor STN (g > 0.46, *p* < 2e-10; Figures 3A,C—detailed breakdown presented in Supplementary Table S1). Although the algorithm-selected currents were similar to the expert settings (Supplementary Table S1), we evaluated the relative efficacy of the contact selection by normalising the calculated VTA overlap with the motor-STN subregion (Figure 3B), and the portion of the calculated VTA within motor-STN boundaries (Figure 3D), dividing with the algorithm-selected currents, and compared them with the corresponding motor STN and VTA fractions when using the expert-selected settings—also normalised by the current indicated in the expert settings. For both comparisons we observed an improvement in terms of the normalised overlaps over the expert settings. The difference was particularly pronounced for the motor STN coverage (Hedges’ g > 0.79, *p* < 2e-20), and smaller for the VTA containment within the motor STN (g > 0.07, *p* < 0.03). The inclusion of clinical review data had a minor influence on these measures (motor STN coverage: g = 0.17, VTA containment: g = 0.19).

### Predicted improvement

To evaluate the potential benefit of the calculated settings on patient outcomes, we compiled a subset of 50 patients with a recorded DBS evaluation using UPDRS-III in the first 3 years after surgery. An improvement ratio was calculated from the difference of UPDRS-III scores with DBS stimulation on vs. DBS stimulation off, normalised by the DBS-off score. To estimate the predicted improvement associated with the calculated settings, we trained a random forest regressor using two predictors: current-normalised motor STN coverage (reflecting contact selection effectiveness), and the stimulation current applied.

The regressor predicted the observed improvements with MSE = 0.0095, R^2^ = 0.692 (Supplementary Figure S1A). Predicted improvement scores were comparable between clinical (median: 39.5%) and algorithm-derived settings (36.2% without clinical review data—39.0% with clinical review data, *n* = 174; see Supplementary Figure S1B). This predicted similarity between clinical and algorithm-derived settings was consistent regardless of whether clinical review data were incorporated, with Hedges’ *g* ranging from 0.05 to 0.08 and Wilcoxon *p*-values between 0.096 and 1 (Supplementary Figure S1B). We noted, however, a minor (g = 0.12) but statistically significant difference (*p* = 0.004) between the predicted improvements of the two algorithm-derived setting sets, with the incorporation of the clinical review information yielding slightly increased predicted improvements, closer to the expert-selected clinical settings.

## Discussion

The method developed here aims to provide a faster and accurate alternative to the trial-and-error approach of manual DBS programming. Following existing guidelines and practices (Picillo et al., 2016), we prioritise monopolar stimulation settings. While the selected contacts could also be employed in bipolar configurations––potentially enhancing electric field steering and expanding target coverage––this would require additional considerations regarding contact polarity. Accurate polarity assignment typically depends on detailed neuroanatomical (through tractography, at minimum) or electrophysiological data, or necessitates further clinical evaluation, which fall outside the scope of this automated approach with minimal data inputs.

### Increased contact selection accuracy

Contact selection performance was evaluated by normalising the overlap between the calculated VTA and the motor STN by the stimulation current, yielding a measure of motor STN coverage per mA. The algorithm-derived settings demonstrated a small but statistically significant increase in current-normalised motor STN activation compared to clinically selected contacts. This suggests that the proposed method identifies more effective contact configurations––at a given implant position––for selectively targeting the motor subregion of the STN.

Similarly, we calculated the motor-STN and VTA overlap as a fraction of the VTA, also normalised with the current. Comparing this variable between manually selected clinical settings and the calculated settings showed that a larger portion of the VTA (per current unit) is contained within the target region (motor STN) when using the calculated settings compared to the manually adjusted settings. This significantly reduces electric field leakage in the milieu, by containing more of the high-intensity area within the target region.

Taken together, the differences in these two variables show better selective activation of the target area (motor STN in this case) when using the calculated contact selection.

### Discrepancies with the clinical settings

Exact matches occurred in only nine implantation instances without clinical review input and in 10 instances when clinical review data were included, due to the procedural difference in contact selection between the two methods. The algorithm, with the settings used in this study, selects up to three sub-contacts out of a maximum of eight (in the case of directional electrodes). The clinicians, however, predominantly select entire (sub-)contact groups, matching the clinical review resolution. Thus, the only perfectly matching cases are limited to non-directional electrodes where up to two (out of four) contacts are selected by both methods.

The algorithm’s current selection strategy aims to achieve a median electric field intensity in the target structure equal to the VTA threshold, optimising stimulation precision. This approach yields similar or better absolute coverage of the motor STN compared to manually adjusted clinical settings, and current-normalised analyses reveal greater efficiency per unit of current. To mitigate potential under-estimation or over-estimation of the current with the automated method and allow further fine-tuning, we provide an additional tool to facilitate exploration of different currents and their effects on STN subregion coverage.

### Comparison with alternative methods

Most recently proposed methods for initial DBS settings optimisation aim to improve the patient outcome directly (Boutet et al., 2021; Qiu et al., 2024; Roediger et al., 2022) or approximate a desired volume of tissue activation (Connolly et al., 2021), using statistics from large patient data pools. By contrast, our method aims to optimise the stimulation of brain structures (STN in this instance) modulating the symptoms of the disease. Among data-driven methods, our approach most closely aligns with the StimFit toolbox (Roediger et al., 2022), which also leverages patient-specific MRI data for automated DBS programming.

Our predicted improvement, predicted to be similar to expert settings based on retrospective analysis, is also comparable with the non-inferiority to standard care achieved by the StimFit solution (Roediger et al., 2023). However, our method introduces a few advantages: it integrates clinical review data when available, explicitly targets the motor subregion of the STN to optimise motor symptom control and operates without the need for pre-trained statistical models––substantially reducing computational and data acquisition demands.

Another closely related anatomy-guided approach is the sweet spot-guided algorithm proposed by Nordenström et al. (2022), which uses Lead-DBS for lead reconstruction and VTA overlap with an empirical sweet spot (derived from prior cohorts) to suggest lead levels, contacts, and effect thresholds for rigidity reduction. While this method provides interpretable suggestions via a Matlab/Lead-DBS-based GUI, it relies on approximations like spherical VTA models for speed, which may affect precision. Our pipeline builds on similar foundations but opts for direct targeting of the motor STN centroid from atlas-based parcellation, relying on OSS-DBS for higher-resolution electric field modelling, and incorporating multi-symptom clinical reviews, enabling further patient-specific fine-tuning without predefined sweet spots.

Other state-of-the-art approaches, such as Bayesian optimisation and particle swarm optimisation, have been proposed to efficiently explore the stimulation parameter space. Bayesian optimisation has been applied to reduce the number of required trials by iteratively minimising rigidity based on prior clinical responses (Louie et al., 2021), while particle swarm optimisation method was designed to address complex electrode configurations with segmented leads, and optimise stimulation amplitudes (Peña et al., 2018).

However, these techniques are primarily outcome-driven and do not currently provide structural substrate-centric metrics or predicted improvement values, limiting the feasibility of direct comparison with our anatomy-based method. Similarly, multimodal biomarker-guided methods, like that of Shah et al. (2023), combine resting/movement-state LFPs with imaging to predict clinical efficacy and side-effect thresholds, achieving ~90% accuracy in optimal contact selection but requiring intraoperative electrophysiological data not routinely available in our workflow. Connectomic-based programming, as in Hines et al. (2024), optimises pathway recruitment using detailed axonal models, yielding 43.5% UPDRS-III improvements, but requires advanced computational resources compared to our simulation-focused approach. fMRI-driven algorithms, such as Santyr et al. (2024), demonstrate non-inferior motor outcomes to standard care in a blinded trial but rely on functional scans, contrasting our emphasis on routine anatomical MRI for broad clinical accessibility.

### Limitations

The method relies on pre-operative and post-operative MRI data. Since the brain structures are directly derived from the pre-operative images and the electrode position is derived from the post-operative scan, the output is sensitive to the time delay between pre- and post-operative inputs. Increased time intervals between the pre-operative MRI scans and the post-operative scans may introduce errors both in electrode localisation relative to the target structures, due to random electrode drifts, as well as changes in the localisation of the brain structures themselves, as a result of ageing or disease progression. This is expected to progressively reduce the accuracy of the method for long-term parameter adjustments, without error corrections for the MRI inputs.

Our method considers two parameters: contact selection, and current selection. Multiple additional parameters have to be assigned by the clinicians, including frequency of stimulation, pulse width/shape, possible interleaved stimulations, and bipolar settings. Most of these parameters are usually preset to well-documented values, and are only changed later in the decision process, if necessary (Picillo et al., 2016). Accordingly, we prioritised the most time-intensive component of initial DBS programming: contact and current selection.

In the case of current estimation, our method targets the centroid of the motor STN subregion while applying predefined lower and upper bounds to ensure clinical plausibility. Thus, the suggested current values should be interpreted as an informed starting point rather than definitive therapeutic settings.

Additionally, in the present study, the input of the clinical review data was weighed equally with the geometry-based calculation. This may be unsuitable if the clinical review information is highly prioritised, for instance, to ensure a minimum stimulation effectiveness or minimising side effects. In such scenarios, increasing the weighting of clinical inputs may enhance the method’s alignment with clinical priorities and patient-specific therapeutic goals. Furthermore, future prospective studies with standardised side-effect logging could correlate non-motor STN coverage with specific adverse effects.

Finally, this study was retrospective in nature, with predicted motor improvement derived from a subset of patients—limited by digitised records availability—who had early UPDRS-III evaluation records following DBS implantation. Although the predicted outcomes are consistent with those reported by comparable methods (i.e., similar to expert settings), a prospective clinical trial will be necessary to validate the effectiveness of this approach with greater certainty.

## Conclusion

This study presents a personalised, interpretable method for DBS parameter selection that leverages routinely acquired anatomical imaging and optionally available clinical evaluation data.

By integrating patient-specific MRI with clinical evaluation data and established DBS modelling tools, we demonstrate a significant improvement in targeting the motor subregion of the STN compared to expert settings, and predict similar improvements in clinical outcomes. This represents a promising patient-specific, low-overhead improvement over common clinical standard practice, aiming to accelerate the initial DBS optimisation steps close to their optimal state for targeting PD motor symptoms.

## Data Availability

The data analysed in this study is subject to the following licenses/restrictions: GDPR restrictions for patient information and MRI scans. Requests to access these datasets should be directed to Daniel Novak, xnovakd1@fel.cvut.cz.
